# Hidden Fungal Diversity of the Precious Mediterranean Red Coral *Corallium rubrum*

**DOI:** 10.1111/1758-2229.70353

**Published:** 2026-04-30

**Authors:** Camille Prioux, Marina Carrasco‐Acosta, Valeria Prigione, Francesco Venice, Romie Tignat‐Perrier, Denis Allemand, Christine Ferrier‐Pagès, Giovanna Cristina Varese

**Affiliations:** ^1^ Sorbonne Université, Collège Doctoral Paris France; ^2^ Unité de Recherche sur la Biologie des Coraux Précieux CSM—CHANEL, Centre Scientifique de Monaco Principality of Monaco; ^3^ Centre Scientifique de Monaco Principality of Monaco; ^4^ Department of Biology Instituto Universitario de Investigación en Estudios Ambientales y Recursos Naturales i‐UNAT, University of Las Palmas de Gran Canaria Las Palmas Spain; ^5^ Mycotheca Universitatis Taurinensis, Department of Life Sciences and Systems Biology University of Torino Turin Italy

## Abstract

Corals maintain complex symbiotic relationships with diverse microorganisms, including fungi, which are often overlooked but represent a critical component of the coral holobiont. This study explores the fungal diversity associated with the tissue and skeleton of the red coral *Corallium rubrum* , a key species in Mediterranean Marine Animal Forests (MAFs). Using a culture‐based approach, we recovered a broad spectrum of fungal diversity, dominated by Ascomycota such as *Penicillium*, *Cladosporium* and *Aspergillus*. The discovery of numerous taxa with known bioactive properties underscores the potential ecological and biotechnological relevance of coral‐associated fungi. At the same time, the presence of species such as *Aspergillus sydowii*, which is considered pathogenic under elevated temperatures, raises concerns about coral vulnerability during increasingly frequent Mediterranean marine heatwaves. These taxa should be further investigated to evaluate their pathogenic potential. Overall, our results expand current knowledge of coral–fungal associations, providing a foundation for future work on their ecological significance, role in coral resilience and potential applications in biotechnology.

## Graphical abstract

This study reveals diverse cultivable fungi associated with the red coral *Corallium rubrum* , highlighting both potentially beneficial and pathogenic species. These findings emphasize the biotechnological relevance and ecological impact of coral‐associated fungi, especially as climate change may increase disease risks. Further investigation into their roles is recommended.

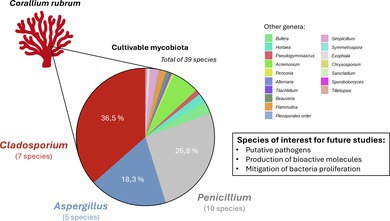

## 1 Introduction

Various microorganisms, including bacteria, archaea, viruses, protists and fungi, are of fundamental importance to marine ecosystems. They drive key processes such as nutrient cycling (Joye et al. 2022) and organic matter decomposition (Moran 2015; Vila‐Costa et al. 2020; Underwood et al. 2022). In addition, they can also establish a symbiotic relationship with different hosts, including corals, and together form a holobiont (Apprill 2017; Dittami et al. 2021; González‐Pech et al. 2024). Among these microorganisms, marine fungi are still largely unexplored, despite their ecological importance, especially regarding their roles in organic matter decomposition, host parasitism and mutualism (Golubic et al. 2005; Raghukumar 2017; Gladfelter et al. 2019; Masigol et al. 2019; Burgaud et al. 2022). This knowledge gap is even more pronounced for fungi associated with corals (Roik et al. 2022), whereas bacterial communities have been extensively studied (van de Water, Allemand, and Ferrier‐Pagès 2018; Voolstra et al. 2024). Fungal diversity in coral holobionts includes both epibiotic and endolithic fungi (Wegley et al. 2007; Amend et al. 2012; Yarden 2014): the former are mainly found in coral tissue, while the latter are able to colonize calcium carbonate skeletons (Kohlmeyer 1969; Kendrick et al. 1982; Golubic et al. 2005).

Coral‐associated fungi fulfil various functions that go beyond pathogenicity (Raghukumar and Ravindran 2012; Roik et al. 2022). While *Aspergillus sydowii* is a well‐documented fungal pathogen associated with aspergillosis of Caribbean Sea fans (Alker et al. 2001; Kim and Rypien 2015; Soler‐Hurtado et al. 2016), many fungi are opportunists rather than direct causative agents of disease (Roik et al. 2022). It has been hypothesized that fungi facilitate nutrient cycling and remineralization, that they produce antimicrobial and chemical compounds which promote coral health and regulate microbial balance, in addition to establishing symbiotic or probiotic relationships with corals, thereby enhancing their resilience to environmental stress (Raghukumar and Ravindran 2012; Roik et al. 2022). Some fungi can act as competitors of endolithic boring algae, potentially mitigating bioerosion under environmental stress (Tribollet and Payri 2001; Golubic et al. 2005). Others have been shown to be involved in calcium carbonate biomineralization through interactions with the organic matrix in different coral species (Le Campion‐Alsumard et al. 1995; Domart‐Coulon et al. 2004). Furthermore, coral‐associated marine fungi have a high biotechnological potential as they produce a variety of bioactive molecules with antibacterial, antifungal, antiviral and anticancer properties (Chen et al. 2022).

Despite these roles, there are significant gaps in our understanding of the diversity and ecological functions of coral‐associated fungi. The study of fungal diversity in corals indeed poses a major challenge when using molecular methods such as metabarcoding, particularly due to host DNA contamination. Some studies report that commonly used genetic markers, like *18S rDNA* and *ITS* regions, result in low recovery of fungal sequences in marine environments (Marcelino and Verbruggen 2016; Góes‐Neto et al. 2020; Prioux, Ferrier‐Pagès, del Campo, et al. 2025), emphasizing the need for complementary approaches, such as culture‐based methods. In addition, fungal communities within corals are often studied without distinguishing between specific compartments (tissue, mucus and skeleton) despite the evidence that these microhabitats may support functionally distinct fungal assemblages (Bonthond et al. 2018; Rabbani et al. 2021). This compartmentalization could have important ecological implications that remain largely unexplored. Finally, most studies have focused on the fungal diversity of tropical corals (Roik et al. 2022), while the mycobiota of deep and temperate corals have been poorly studied (Galkiewicz et al. 2012; Marchese et al. 2021) despite their importance as ecosystem engineers, particularly in the Mediterranean Sea where they built Marine Animal Forests (MAFs; Rossi et al. 2017). The majority of these corals are octocoral gorgonians increasingly threatened by climate change and marine heat waves (Gómez‐Gras et al. 2021; Garrabou et al. 2022; Estaque et al. 2023). One of them, the red coral *Corallium rubrum* (Linnaeus 1758), not only has an ecological function in the MAFs but also holds significant cultural and economic value, having been harvested for centuries for use in jewellery (Santangelo and Abbiati 2001; Tsounis et al. 2010).

Previous research has extensively characterized the bacterial microbiome (van de Water et al. 2016, van de Water, Allemand, and Ferrier‐Pagès 2018; Prioux et al. 2024), and to a lesser extent, the eukaryome (microeukaryotic fraction of the microbiome) of *C. rubrum* (Prioux, Filipponi, et al. 2025). Recent studies are even beginning to connect the health decline of *C. rubrum* during marine heatwaves (MHWs) to the presence of pathogenic or opportunistic bacteria, as well as certain microeukaryotes (Prioux et al. 2023; Prioux, Ferrier‐Pagès, del Campo, et al. 2025; Prioux, Ferrier‐Pagès, Lamarca, et al. 2025). However, the fungal community associated with this species remains largely unexplored and poorly understood. With this work, we aim to expand our knowledge of the fungal community associated with healthy‐looking *C. rubrum* colonies and potentially our understanding of coral–fungi interactions in temperate zones using culture techniques. This study provides an essential baseline for monitoring changes in fungal communities due to climate change and for guiding future conservation efforts.

## 2 Material and Methods

### 2.1 Samples Collection

Six multibranched colonies of *C. rubrum* (7–8 cm in length) were collected at a depth of 40 m in Villefranche‐sur‐Mer, France, under the authorization of the Direction Inter‐Régionale de la Mer Méditerranée. Following collection, samples were transported in seawater to the aquarium facilities of the Centre Scientifique de Monaco (10 km from Villefranche‐sur‐Mer), where they were maintained in two 20 L aquaria supplied with a continuous flow of Mediterranean seawater pumped at 40–50 m depth in front of the laboratory, filtered through a 5 μm charcoal filter and treated with UV light. Nubbins were then transported in aquarium seawater and processed at the laboratory facilities of the Mycotheca Universitatis Taurinensis (see section below).

### 2.2 Fungal Isolation

Six coral nubbins were subjected to serial sonication (three times for 30 s each) in sterile seawater to remove debris and microorganisms not strictly associated with the red coral. For each nubbin, the soft tissue was separated from the skeleton using sterile scalpels and tweezers and then homogenized in 4 mL of sterile seawater using a Sterilmixer II homogenizer (PBI International, Milan, Italy). The resulting solution was diluted 1:50 (w/v) in sterile seawater. The remaining skeletons were sonicated five more times for 60 s to ensure complete removal of residual soft tissue. Each skeleton was crushed through a sterile mortar with 4 mL of sterile seawater, and the resulting solution was diluted 1:10 (w/v) in sterile seawater. The soft tissue and skeleton mixtures were used for culturomics, spreading 1 mL onto 15 cm diameter Petri dishes. Four different growth media were used to maximize fungal isolation: Corn Meal Agar Sea Water (CMA‐SW, 2 g corn meal, 15 g agar in 1 L of seawater); Gelatin Agar Sea Water (GA‐SW, 20 g gelatin, 15 g agar in 1 L of seawater) to mimic the composition of the organic material rich in collagen; Pimaricin Corn Meal Agar Sea Water (P‐CMA‐SW, consisting of CMA‐SW supplemented with 5 mg/L pimaricin, 250 mg/L ampicillin, and 10 mg/L rifampicin), selective for Stramenopiles and other fungus‐like organisms; Coral Agar Sea Water (CA‐SW, containing homogenized 18 g coral soft tissue and skeleton, and 20 g agar in 1 L of seawater), a medium designed to mimic coral conditions. To prevent bacterial growth, all media were supplemented with an antibiotic mix (Gentamicin sulfate, 40 mg/L; Piperacillin and Tazobactam, 11 mg/L).

Five replicates were prepared for each medium and tissue type (soft tissue and skeleton). Plates were incubated in the dark at 15°C for 6 weeks and at 25°C for an additional 6 weeks. During the incubation period, plates were constantly monitored for fungal growth. Fungal isolates that developed were transferred to axenic cultures and taxonomically identified.

### 2.3 Fungal Identification

Fungi were identified using a polyphasic approach combining morpho‐physiological observation with molecular analyses. DNA was extracted using the DNeasy PowerBiofilm kit (QIAGEN, Hilden, Germany), with modifications to enhance efficiency as described by Prioux et al. (2023). Specific primers were used to amplify DNA markers for each fungal genus identified during the morpho‐physiological observations. When the initial amplification did not allow species‐level identification on blastn (NCBI; https://blast.ncbi.nlm.nih.gov), a second round of amplification was performed with an alternative primer set, as detailed in File S1. All sequences are available in File S1 and fungal strains are preserved at the Mycotheca Universitatis Taurinensis (MUT, http://www.mut.unito.it), University of Turin, Italy.

### 2.4 Statistical Analyses

The number of Colony Forming Units (CFU) per gram of dry weight (CFU g‐1 dry weight) was calculated both per condition and per fungal species.

To estimate the dry weight of coral samples, fresh biomass was measured by separating soft tissue from the skeleton. A drying ratio was determined using preliminary lyophilization experiments conducted on three additional coral fragments, which were weighed before and after 24 h of lyophilization. This ratio was then applied to estimate the dry weight of the experimental samples. This approach allowed us to derive a normalized biomass measure comparable across tissue types. All calculations and raw measurements are detailed in File S1. Statistical analyses were conducted in the R environment (version 4.2.1). Differences in abundance (CFU/g dry weight) were assessed using the Kruskal–Wallis test, considering tissue type, isolation temperature and media as independent factors. To evaluate differences in mycobiota composition across culture media, tissue types and isolation temperatures, we employed a permutational multivariate analysis of variance (PERMANOVA), with significance determined using a pseudo‐F statistic and a *p* value threshold of < 0.05. Principal Coordinate Analysis (PCA) was used to visualize data.

## 3 Results and Discussion

Coral‐associated fungi play pivotal roles within the coral holobiont, not only by supplying essential compounds and offering protection against pathogens but also, in some cases, acting as pathogens themselves. However, fungal communities remain largely understudied compared to other coral‐associated microorganisms (Roik et al. 2022). As corals face increasing anthropogenic stress, understanding the diversity and ecological functions of these fungi is crucial for advancing coral health research and guiding effective conservation efforts. Moreover, fungi isolated from corals are emerging as valuable sources of bioactive natural products, producing a diverse array of compounds with significant promise for drug discovery and biomedical applications (Said et al. 2018; Hou et al. 2019; Ma and Qi 2019; Chen et al. 2022).

### 3.1 Optimizing Isolation Strategies: Influence of Growth Media, Temperature and Tissue Type

**FIGURE 1:**
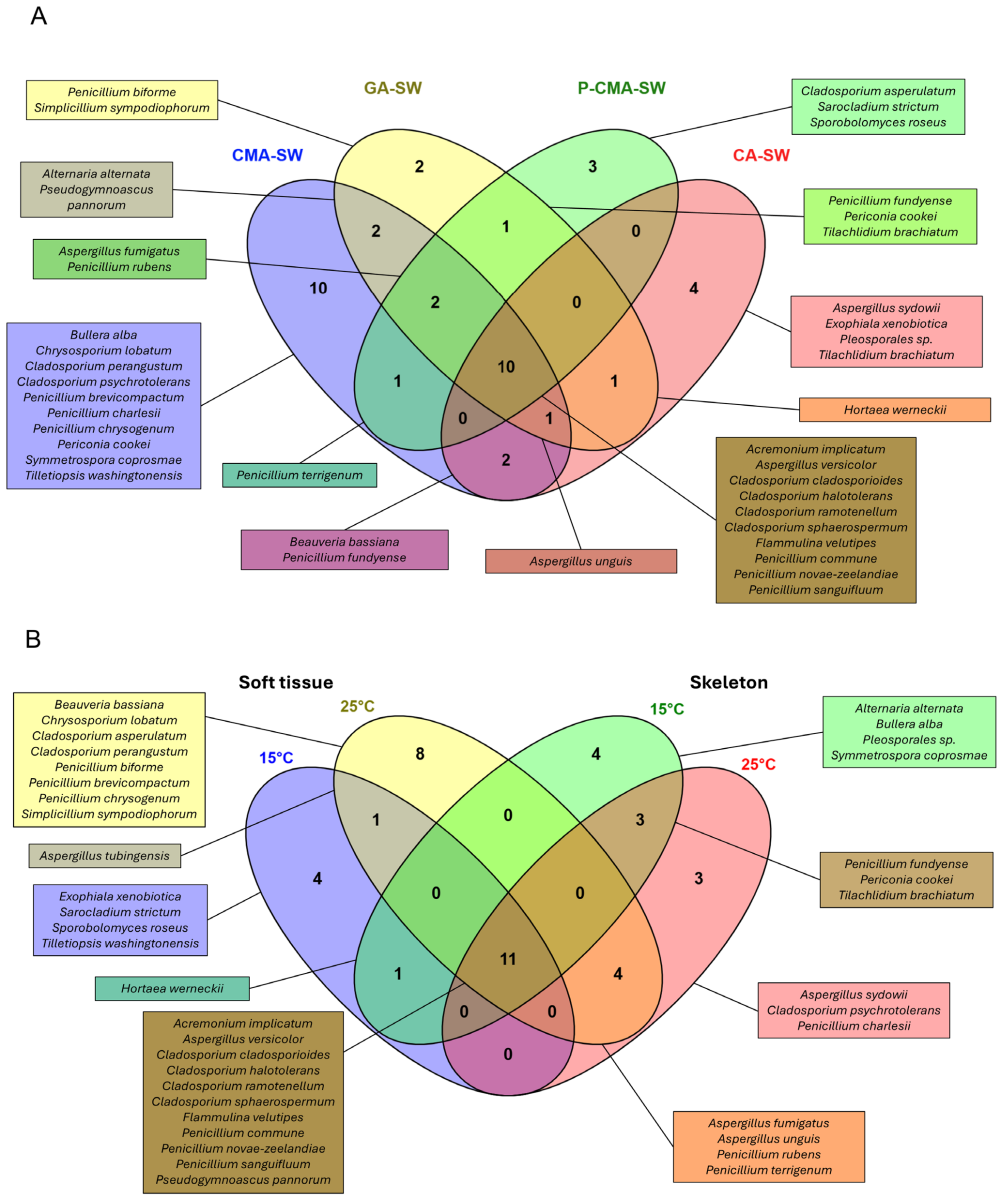
Influence of the isolation techniques on the number of culturable fungal species isolated from *Corallium rubrum* . Details on the number of exclusive and common species found in the different growth media (A) and the two coral tissues (soft tissue and skeleton) at different incubation temperatures (15°C and 25°C) (B).

Overall, 39 taxa were isolated from the red coral *C. rubrum* (File S1). The use of two different incubation temperatures and four growth media had a significant impact on the yield and diversity of cultivable fungi. On the 39 species isolated, 20 were obtained from at least two different media (Figure 1A), but media‐based patterns were also observed. The composition of isolation media is a critical determinant of the fungal community recovered from marine environments (Foster and Bills 2011; Overy et al. 2019; Zhang et al. 2024). Remarkably, 25.6% of the taxa isolated in this study were exclusive to CMA‐SW, a medium that is widely used for fungal cultivation. CMA‐SW is enriched in complex carbohydrates, which selects for fast‐growing, highly sporulating genera such as *Aspergillus* and *Penicillium* (Zhang et al. 2024). By contrast, another nutrient‐rich medium, GA‐SW, formulated with gelatin to emulate the collagen‐rich organic matrix of red coral (Roy et al. 2021), did not recover any taxa that were not also present on the other media. This absence of exclusive isolates suggests that the gelatin matrix did not confer a selective or niche‐specific advantage capable of capturing fungi that would otherwise be out‐competed on more generic media. Interestingly, the CA‐SW medium, which incorporates homogenized coral tissue and skeleton to replicate the natural host environment, recovered 10.3% of its isolates as media‐specific taxa. This result indicates that the micro‐environmental cues present in coral tissues, such as specific protein substrates, ion concentrations or micro‐aerobic conditions, may selectively stimulate the growth of fungi that are either specialized to, or better adapted for the coral niche. The selective P‐CMA‐SW medium was formulated to favour the recovery of less competitive taxa, yielding 7.69% of isolates that were unique to this substrate, organisms that would otherwise have been out‐competed on broader, nonselective media. By integrating general, nutrient‐rich, selective and host‐mimicking growth media, we tried to mitigate as much as possible the risk of medium‐specific bias to obtain a more exhaustive representation of the marine fungal assemblage present in our samples. The results demonstrated the critical role of media selection in marine mycological research.

While 17.9% of taxa were exclusively isolated at 15°C (psychrotolerant), a larger portion (41%) grew only at 25°C (mesophilic) (Figure 1B), suggesting that many fungal strains display mesophilic/thermophilic tendencies. Even if complementary studies are needed, specifically assessing the impact of thermal stress on the fungal communities, our results suggest that mesophilic and thermotolerant or thermophilic taxa could become prevalent under thermal stress conditions. By expanding the range of incubation temperatures in our cultures to 25°C, we were able to isolate a broader diversity of fungi, including taxa that require temperatures above 15°C to thrive. This finding is particularly significant, as it highlights a tendency toward thermotolerance within the fungal community, a potential warning sign that warmer conditions may increasingly favour the proliferation of these species. Some studies, mainly on tropical hexacoral species, showed that high temperatures correlate with increased fungal diversity and abundance (Amend et al. 2012; Thurber et al. 2009), with some species being involved in coral skeleton bioerosion and diseases (Bentis et al. 2000; Gleason et al. 2017; Góes‐Neto et al. 2020). The thermophilic taxa recovered in this study may represent opportunistic or pathogenic agents of *C. rubrum* , proliferating under conditions of thermal stress, and their increasing presence could indicate an ongoing reshaping of the microbial community toward more thermotolerant, and potentially more pathogenic, species. Investigating these organisms is of particular urgency in the face of increasingly frequent and severe MHWs in the Mediterranean Sea, where sea‐surface temperatures are rising 20% faster than the global average (Oliver et al. 2018; Darmaraki 2019). Recurrent MHWs have produced record‐breaking temperature anomalies that have precipitated widespread coral mortality, including documented mass‐mortality events in *C. rubrum* (Cerrano et al. 2000; Huete‐Stauffer et al. 2011; Verdura et al. 2019; Piazzi et al. 2021; Garrabou et al. 2022). Elucidating the ecological roles of these thermophilic taxa could clarify whether they are simply biomarkers of dysbiosis or active drivers of the disease phenotype that repeatedly affects red coral following MHWs, thereby informing targeted management and conservation strategies.

Overall, 15 taxa were isolated from both tissue types, but 13 species were exclusively retrieved from soft tissue and 10 species were found exclusively in the skeleton (Figure 1B). The observed differences in fungal communities between the soft tissues and skeleton of red coral likely arise from distinct microenvironmental conditions of each habitat. The soft tissues are directly exposed to seawater and are rich in oxygen and host‐derived nutrients such as protein, carbohydrates and lipids (Rossi and Tsounis 2007), creating a dynamic environment that allows for colonization by a diverse array of microbes. In contrast, the skeleton represents a more stable but nutrient‐poor environment, predominantly composed of magnesium‐rich calcite and organic matter (Grillo et al. 1993), with lower oxygen availability, favouring microbes that are specialized to survive under oligotrophic or even anoxic conditions, and that may participate in mineral cycling or bioerosion. These differences in abiotic conditions and host interactions act as strong selective pressures, leading to the presence of ‘soft tissue specialists’ and ‘skeleton specialists’.

**FIGURE 2:**
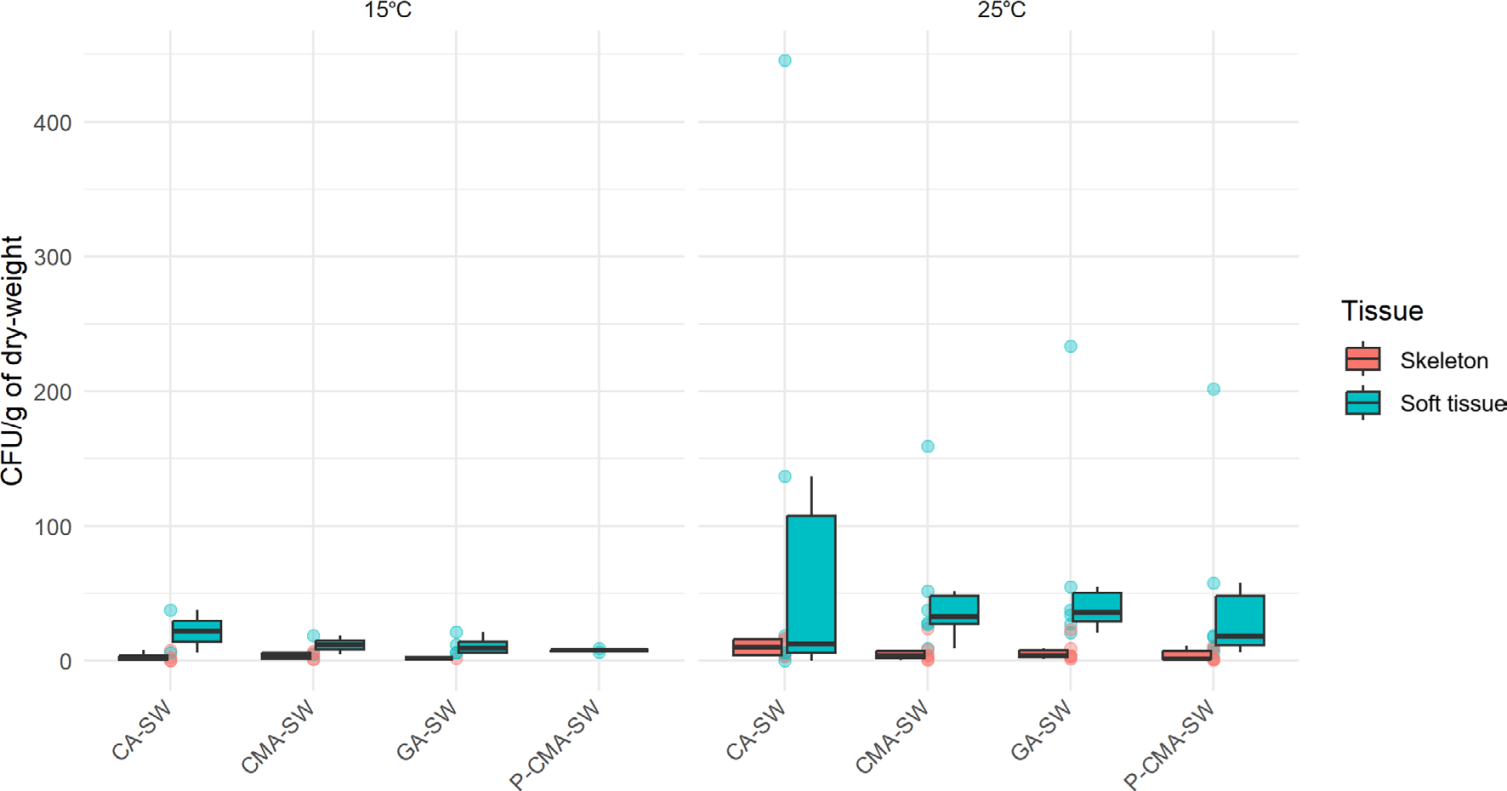
Composition of the mycobiota of *Corallium rubrum* . Colony‐forming unit (CFU) per gram of skeleton or soft tissue dry weight across isolation temperatures and growth media.

Quantitative analysis revealed no significant effect of host tissue type, incubation temperature or growth medium on the number of fungal isolates recovered (Colony‐forming units per gram of dry weight; Kruskal–Wallis, *p* > 0.05; Figure 2; File S2). In contrast, multivariate analysis of community composition demonstrated clear shifts associated with both tissue origin and temperature (PERMANOVA, *p* = 0.001 for tissue type, *p* = 0.052 for temperature; File S2). PCA further illustrated that most samples clustered tightly, whereas a subset of isolates obtained from soft tissue incubated at 25°C formed distinct outliers, thereby driving the significant PERMANOVA signal (File S2). Given the exploratory and descriptive nature of the study, and the limited sample size, these results should be interpreted with caution. However, these findings suggest that while the overall culturable fungal biomass may be similar across sample types, the mycobiota is sensitive to both host tissue specialization and incubation conditions. The pronounced divergence observed in the 25°C soft‐tissue isolates implies that elevated temperatures may selectively favour particular taxa or alter growth dynamics, underscoring the importance of temperature and tissue context in shaping culturable fungal communities.

### 3.2 Diversity and Functional Potential of Isolated Fungal Species From *C. rubrum*

The polyphasic approach allowed the identification of 38 on 39 isolated fungi at the species level, with the exception of a single strain (*Pleosporales* sp.; File S1). The fungal communities associated with the soft tissue and skeleton of *C. rubrum* were predominantly composed of Ascomycota (34 taxa; 87.2%), while Basidiomycota accounted for 12.8% (five taxa), a distribution pattern commonly observed in marine environments compared to terrestrial environments where Basidiomycota are generally more represented (Jones et al. 2015; Bärlocher and Boddy 2016). Ascomycota are known to dominate the fungal communities associated with corals, often exceeding 85% in relative abundance across various studies, with Sordariomycetes being the most frequently reported class (Roik et al. 2022). It can be hypothesized that marine Ascomycota may assume ecological functions in marine habitats that are often associated with Basidiomycota in terrestrial ecosystems, including the breakdown of lignocellulosic compounds (Panno et al. 2013; Raghukumar 2017; Balabanova et al. 2018) and hydrocarbons (Garzoli et al. 2015; Bovio et al. 2017). On the other hand, it cannot be excluded that the low presence of Basidiomycota observed derives from the intrinsic biases in the isolation techniques commonly applied in studies on marine fungi.

Among the identified genera, *Penicillium* (10 species), *Cladosporium* (seven species), and *Aspergillus* (five species) were the most diverse (Figure 1). These ubiquitous genera appear across a wide range of marine matrices, routinely detected in sediments, seawater and as part of the microbiota of sponges, corals and other invertebrates (Richards et al. 2012; Bovio et al. 2018, 2019; Gladfelter et al. 2019; Roik et al. 2022; Schultz et al. 2022), indicating that they can survive in both particulate and free‐water habitats. Their presence in multiple compartments raises the question of whether they participate in cross‐habitat interactions. For example, some *Cladosporium* species observed in diseased tropical corals from La Réunion Island were also detected in the surrounding seawater and sediment (Stenger et al. 2024), suggesting potential interactions and exchanges between the different ecological compartments. In future studies, it would be particularly interesting to also attempt the isolation of fungi from the seawater directly surrounding the corals in order to better understand the dynamics, sources, and potential exchanges of fungal communities between the host and its environment.

Despite the frequent associations of these genera with corals (Amend et al. 2012; Roik et al. 2022), our findings from *C. rubrum* tissues demonstrate that the diversity of fungal communities in corals remains largely unexplored. To our knowledge, there are no previous studies that have isolated fungal strains from Mediterranean corals (Literature review; File S3), making the diversity uncovered here especially noteworthy and highlighting the need for further research on these fungal communities. Notably, among the species observed in *C. rubrum* , six of *Penicillium* species (*P. novae‐zeelandiae*, *P. fundyense*, *P. charlesii*, *P. biforme* , *P*. *sanguifluum* and *P. brevicompactum*) and three of *Cladosporium* (*C. perangustum*, *C. asperulatum* and *C. psychrotolerans* ) were never reported in other coral species (File S3). Some members of these genera, whose role in coral ecology is still obscure, are known to be producers of a wide range of bioactive metabolites, making them important targets for natural product discovery (Chen et al. 2022). For instance, species like *Cladosporium halotolerans*, *Cladosporium cladosporioides* and *Aspergillus* *versicolor* , isolated from corals, produce bioactive molecules such as polyketides, compounds that exhibit cytotoxic effects against certain types of cancer cells (Zhuang, Teng, Wang, Liu, et al. 2011, 2011; Espinoza et al. 2021; Wang et al. 2021). Besides, a strain of *Aspergillus unguis* isolated from the tropical coral *Pocillopora damicornis* and recovered in the red coral as well, was shown to produce antiosteoclastogenic molecules (Zhang et al. 2022).

Several melanized fungal species identified in this study, including *Hortaea werneckii*, *C. cladosporioides*, *Cladosporium sphaerospermum*, *Aspergillus fumigatus*, *A. versicolor* and *Alternaria alternata* , have been shown to produce protective pigments and bioactive compounds, such as melanin, carotenoids and mycosporine‐like amino acids, that may contribute to UV protection in the coral holobiont (Volkmann et al. 2003; Kogej et al. 2006; Avalos and Carmen Limón 2015; Wong et al. 2019; Roik et al. 2022). However, as *C. rubrum* is a sciaphilous species, mainly observed in dark environments such as cave entrances, overhangs and crevices (Ballesteros 2006), its role in the red coral remains to be fully elucidated.

Interestingly, previous studies on other coral species have shown that some of the fungi from this study isolated at 25° can exhibit antibacterial activity. This is the case of *A. versicolor* isolated from the coral *Hemicorallium imperiale*, a member of the same family (Coralliidae) as the red coral (Dong et al. 2023), *A. unguis* from the seagrass *Thalassia hemprichii* (Setyati et al. 2023), *A. fumigatus* from diverse Red Sea corals (Abd El‐Rahman et al. 2020) and *Penicillium rubens* from an unidentified coral species (Ying, Li, Wang, et al. 2023; Ying, Li, Yang, et al. 2023). In detail, they produce antibacterial compounds such as rubensteroids and polyketides, which exhibit inhibitory effects against *Vibrio harveyi* , *Vibrio alginolyticus* and *Vibrio parahaemolyticus* . These *Vibrio* species are known for their pathogenicity in corals when seawater temperature exceed 22°C (Vezzulli et al. 2010; Tout et al. 2015). They were observed to proliferate in the red coral during MHW events (Prioux et al. 2023; Tignat‐Perrier et al. 2023). Therefore, it can be hypothesized that such fungi may play a protective role for corals by limiting the proliferation of pathogenic bacteria during MHWs. However, two *Cladosporium* species among tissue specialists isolated at 25°C (*C. perangustum*, *C. esperulatum*) are terrestrial plant pathogens (Jang et al. 2013; Liu et al. 2017; Temperini et al. 2018; Hu 2023; Chen et al. 2024) often retrieved in marine environment, but their role in coral microbiome and health is still unknown.

Skeleton specialists included three taxa isolated exclusively at 25°C: *A. sydowii, C. psychrotolerans*, and *P. charlesii*. While the two last have never been observed in corals before, *A. sydowii* is a well‐known coral fungus, observed in at least 24 coral species including nine octocoral species (File S3). This fungus is a known pathogen of *Leptogorgia* spp. from the Ecuadorian Pacific (Soler‐Hurtado et al. 2016) and of Caribbean fans of the *Gorgonia* genus (Alker et al. 2001; Kim et al. 2015), being responsible for the coral ‘aspergillosis’, which causes severe tissue lesions and leads to mass mortality (Smith et al. 1996; Nagelkerken et al. 1997; Toledo‐Hernández et al. 2008). Although its role in *C. rubrum* is still unclear, its presence in the skeleton‐associated fungal community and its proliferation at 25°C raise concern about its potential to develop under MHW conditions and its possible impact on coral health and resistance.

Of the 15 species retrieved from both compartments, 11 species were consistently detected across all temperature conditions, suggesting a broad ecological plasticity (Figure 1B). The four *Cladosporium* species have been previously reported in association with a wide range of coral hosts (24 species in total, including 10 octocoral species; File S3) supporting their role as widespread generalists within the coral fungal community. *C. halotolerans* , in particular, has been found to have beneficial effects on corals (Granit et al. 2025). Specifically, this fungus may help corals cope with heat stress by reducing tissue loss, lowering stress gene expression, and supporting higher photosynthetic efficiency in algal symbionts (Granit et al. 2025). *A. versicolor* and *Penicillium commune* are also widely distributed among corals, especially *A. versicolor* (observed in 28 coral species, including 10 octocoral species; File S3) and likely play a significant ecological role in the coral holobiont. When isolated from corals, these species have been shown to exhibit a broad range of bioactivities, including antioxidant, radical‐scavenging, antimicrobial and antifouling activity (Zhuang, Teng, Wang, Liu, et al. 2011; Chen et al. 2012; Wang et al. 2012; Zhang et al. 2019).

On the other hand, the other consistently detected species were never reported in coral fungal communities to our knowledge (File S3). One of them, *Pseudogymnoascus pannorum*, has already been isolated from marine animals thriving in Antarctica (Godinho et al. 2019; Ogaki et al. 2019) or from bivalves (Borzykh and Zvereva 2015). Their repeated occurrence in all compartments and temperature conditions in this study could indicate potentially relevant specific associations with the red coral. Indeed, their potential to produce secondary metabolites (Fukushima‐Sakuno 2020) and to exert antioxidant (Henríquez et al. 2014) and antimicrobial activities (Zhang et al. 2019, 2021) could be of great importance for the holobiont. These findings raise questions about the functional roles of these fungi and whether their persistence under varying environmental conditions contributes to coral holobiont stability.

Finally, it is worth noting the isolation, from both soft tissue and skeleton, of *H. werneckii*, a halophilic black yeast, causative agent of human skin infection ‘Tinea Nigra’ occurring in tropical and subtropical regions of the world and reported as a cause of occasional marine fish disease (Todaro et al. 1983; Bonifaz et al. 2008). *H. werneckii* was isolated for the first time from the Mediterranean Sea a few years ago (Leo et al. 2019). The presence in coral of this species with a pathogenic aptitude is, therefore, worthy of attention.

## 4 Conclusion

In conclusion, our findings support the idea that culture parameters, such as temperature and media selection, play crucial roles in uncovering the hidden diversity of cultivable fungi associated with corals. The culture‐based approach in this study proved effective at recovering fungal diversity and emphasizes the continued relevance of culturing methods in marine microbial ecology. However, this study focused on six coral colonies from a single locality, constraining the generalizability of our findings and precludes assessment of biogeographic or population‐level variation in mycobiota composition. Overall, 61% of the isolated fungal species (24 species) were reported here for the first time in association with corals, underscoring the largely unexplored diversity of coral‐associated fungi. The discovery of both generalist taxa, which may occur across multiple coral hosts or environmental contexts, and specialist taxa, which appear more tightly associated with *C. rubrum* , points to a complex and structured mycobiota. This structure is likely shaped by a combination of host‐derived selection pressures, such as the distinct physicochemical characteristics of coral compartments (soft tissue and skeleton), and environmental conditions, including temperature, nutrient availability and local microbial interactions. The fungal diversity observed in this study, along with findings reported in scientific literature, and the compartmentalization of the fungal community within the coral, may reflect ecological selection processes that potentially support coral function and enhance their ability to adapt to environmental stresses. The finding of some known pathogens of other coral species and with a thermotolerance attitude warrants further investigation. A focused experimental approach could involve inoculating healthy coral fragments with the isolate under controlled, temperature‐elevated conditions that mimic marine heat‐wave scenarios. Concurrent visual assessment of coral tissue integrity, coupled with transcriptomic profiling of the pathogen under ambient and stressed temperatures, would provide complementary data to discern whether the fungus actively contributes to disease progression or merely colonizes the host opportunistically during thermal perturbations. Furthermore, the identification of certain fungal species, from which biomolecules of ecological and biotechnological interest have been described, creates opportunities for future studies focused on the valorization of marine fungi‐derived biomolecules with potential applications in several industrial sectors. Overall, this study highlights the pressing need to further investigate the largely unexplored mycobiota of Mediterranean octocorals in order to gain a comprehensive understanding of their potentially beneficial or detrimental roles for their hosts, particularly in the context of ongoing climate change.

## Author Contributions

**Camille Prioux:** writing – original draft, writing – review and editing, investigation, formal analysis, conceptualization, visualization, software, data curation. **Christine Ferrier‐Pagès:** conceptualization, investigation, validation, writing – review and editing, project administration, supervision, resources. **Francesco Venice:** writing – review and editing, validation, methodology, software, data curation, formal analysis. **Denis Allemand:** writing – review and editing, validation, project administration, resources, funding acquisition. **Giovanna Cristina Varese:** conceptualization, investigation, funding acquisition, methodology, validation, writing – review and editing, project administration, supervision, resources. **Valeria Prigione:** conceptualization, investigation, validation, writing – review and editing, data curation, supervision, resources. **Romie Tignat‐Perrier:** conceptualization, writing – review and editing, validation, project administration, supervision, formal analysis. **Marina Carrasco‐Acosta:** conceptualization, investigation, writing – review and editing, validation, methodology, data curation, supervision.

## Conflicts of Interest

The authors declare no conflicts of interest.

## Supporting information


**File S1:** Methodological approach for the identification of fungal strains cultured from the red coral.

**File S2:** Results of the statistical tests.

**File S3:** Literature review of fungal species isolated from various coral hosts.

## Data Availability

The data that supports the findings of this study are available in the Supporting Information of this article.
